# Use of sequence barcodes for tracking horizontal gene transfer of antimicrobial resistance genes in a microbial community

**DOI:** 10.1093/ismeco/ycaf113

**Published:** 2025-07-10

**Authors:** Veera Partanen, Svjetlana Dekić Rozman, Antti Karkman, Johanna Muurinen, Teppo Hiltunen, Marko Virta

**Affiliations:** Department of Microbiology, University of Helsinki, Viikinkaari 9, 00790, Helsinki, Finland; Department of Microbiology, University of Helsinki, Viikinkaari 9, 00790, Helsinki, Finland; Department of Microbiology, University of Helsinki, Viikinkaari 9, 00790, Helsinki, Finland; Department of Microbiology, University of Helsinki, Viikinkaari 9, 00790, Helsinki, Finland; Department of Biology, University of Turku, Turku,Vesilinnantie 5, 20014, Finland; Department of Microbiology, University of Helsinki, Viikinkaari 9, 00790, Helsinki, Finland

**Keywords:** horizontal gene transfer, epicPCR, sequence barcode, experimental evolution, antimicrobial resistance

## Abstract

One of the most important knowledge gaps in the antimicrobial resistance crisis is the lack of understanding regarding how genes spread from their environmental origins to bacteria pathogenic to humans. In this study our aim was to create a system that allows the conduction of experiments in laboratory settings that mimic the complexity of natural communities with multiple resistance genes and mobile genetic elements circulating at the same time. Here we report a new sequence-based barcode system that allows simultaneous tracking of the spread of antimicrobial resistance genes from multiple genetic origins. We tested this concept with an experiment in which we added an antimicrobial resistance gene to different genetic environments in alive and dead donors and let the gene spread naturally in an artificial microbial community under different environmental conditions to provide examples of factors that can be investigated. We used emulsion, paired-isolation, and concatenation polymerase chain reaction to detect the new gene carriers and metagenomic analysis to see changes in the genetic environment. We observed the genes moving and were able to recognise the barcode from the gene sequences, thus validating the idea of barcode use. We also saw that temperature and gene origin had effects on the number of new host species. Our results confirmed that our system worked and can be further developed for more complicated experiments.

## Graphical Abstract

**Graphical Abstract:**
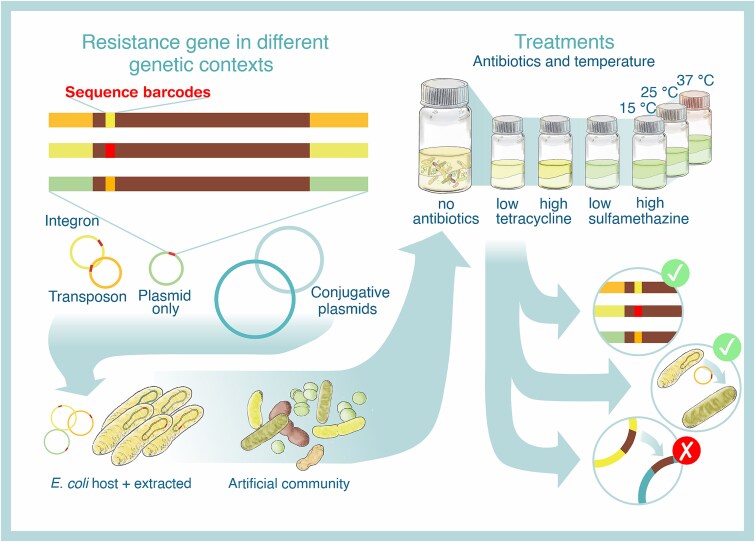

## Introduction

In recent years, multiple reports and predictions have painted a dark picture of the antimicrobial resistance (AMR) crisis and our future with it (most notably, see [1, 2]). AMR is, however, a natural phenomenon, with resistance becoming a problem mostly in human pathogens when the infections they cause cannot be treated [3, 4]. Many antimicrobial resistance genes (ARGs) originate from environmental bacteria [3, 5], which can also operate as a resistance reservoir [6], from which the ARGs can spread to the problematic human-associated species [5]. The process of transmission of ARGs from environmental reservoirs to human-associated species and finally to pathogenic bacteria is one of the most important knowledge gaps in the understanding of the evolutionary mechanisms that lead to the emergence of healthcare nightmares such as those recently reported [7–9].

Bacteria can become resistant towards antimicrobials through de novo mutations or by obtaining new genetic material through horizontal gene transfer (HGT). The mobile genetic elements (MGEs) are particularly problematic as they can contribute to new pathogen outbreaks [10–12]. MGEs such as plasmids with multiple ARGs can make a strain multiresistant in a single transfer event. It is also suspected that MGEs can transmit the ARGs between ecological niches [13] and between bacteria in more pristine and anthropic environments and thus introduce new genes to the problematic pathogen gene pool [4]. In addition to plasmids, MGEs include transposons, which themselves can be inside other MGEs and efficiently change the genetic environment of ARGs [14]. Integrons are not mobile per se, but they can often be found inside other MGEs where they can collect the ARGs for storage in the mobilome (collection of mobile genetic elements) [15].

The HGT networks of ARGs are a complicated research topic. Some approaches to investigate them and factors affecting them have included network analysis from metagenomes [13, 16], following single plasmids in experimental systems [17–19], and, to some extent, comparing ARG hosts at two different stages along a (wastewater purification) process. [20]. Metagenome analysis provides a lot of data, but not about how the genes have been spreading, which bacteria were their original hosts, or what factors were affecting the process. Comparison of ARG hosts along a process (e.g., [20]) could possibly elucidate HGT. However, one cannot entirely rule out the possibility that the ARG-carrying bacteria were present to begin with and the changes observed in the carrier pool were due to abundance changes around the detection threshold. Still, both of these approaches (metagenomes and comparing ARG hosts along a process) enable study of the actual phenomena happening in the environment, unlike experiments in the laboratory. Then again, conducting a laboratory experiment with a single plasmid gives more detailed information about the HGT, as the original host is known and the new hosts can be identified. This approach also allows controlling and studying the factors that influence the transfer of the ARGs. However, when working with a single plasmid, capturing the influential factors could be dependent on the plasmid chosen and its host species. For example, some *Aeromonas* plasmids are already rearranging at temperatures slightly above 20°C [21], while *Escherichia coli* plasmids are still stable at their host’s optimal temperature of 37°C. Therefore, it would seem logical that these plasmids react differently to temperature changes. Then again, it would be time-consuming if one had to repeat the same experiments with several plasmids.

To overcome some of these restrictions for researching HGT in more natural and more complicated settings, we created a sequence–barcode based system, which together with emulsion, paired-isolation, and concatenation polymerase chain reaction (epicPCR [22]) allows us to always know the origin of the gene and track resistance genes from different genetic environments and hosts simultaneously. EpicPCR is a single-cell, free of culturing, droplet PCR method, in which the gene of interest is fused with (usually) the 16S rRNA gene of the host to find the species carrying the gene. Previously epicPCR has been used, for example, to find hosts of antibiotic resistance genes in wastewaters [20, 23], research virus–host interactions [24], and identify bacterial groups taking part in sulphate reduction [22, 25]. We sought to find the barcodes in epicPCR sequences to recognize the new host species and in metagenome sequences to see whether the genes had changed their genetic environment and become mobile. Our objective was to create and test a system that in the future allows research of more complicated HGT systems with complicated communities and multiple ARGs and genetic environments. As an example of factors that can be studied with our system, we chose temperature, because geographically, there seems to be a positive correlation between temperature and higher antibiotic resistance load [26, 27]. As another example, we chose two antibiotics with lower than minimum inhibitory concentrations (MIC), which has been shown to cause selection pressure [28] and increase the number of recipient strains of HGT [18, 29]. We also included six different origins with three different genetic environments, including integron and transposon, to allow mobilisation of our gene of interest. Integrons and transposons are important carriers and storage entities for ARG [14]. The aim of this study was to understand factors affecting HGT and thus contribute to global efforts against AMR.

## Materials and methods

### Bacterial strains and plasmids

An artificial bacterial community was put together (the full list of species can be found in Table S1) from strains able to grow in similar laboratory conditions and genera that were found to be common ARG carriers in Viikinmäki wastewater treatment plant in an earlier study [20, 30]. The community was used as the receiving community in the microcosm experiment. As the donor or original host species for the resistance genes *E. coli* JE2571 was used.

A resistance gene, *sul1*, which confers resistance to sulphonamide class antibiotics, was chosen based on its mechanism of function. The product of *sul1* replaces a sensitive target protein inside the cell with another, whose function the antibiotic cannot inhibit. Instead of degrading the antibiotic and protecting other cells around the host, the use of this gene should give a selective advantage only to the cell carrying the gene, thus allowing the gene to persist in the community.

**Figure 1:**
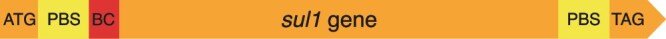
Structure of the modified *sul1* gene used in the experiments. The gene starts with its normal ATG start codon, which is immediately followed by forward primer binding site (PBS) and barcode (BC) sequences. After that the gene continues normally until reverse primer binding site followed by stop codon TAG.

Artificial primer binding sites (forward: GGTCGTGAGCACCTAGGGTCTCATG; reverse: GGGCAGAGCCTCAGAACACTT) were added to the resistance gene inside the reading frame (Fig. 1) to be used in epicPCR to ensure the amplification of the modified gene only, and, in the metagenomic analysis, to help distinguish the gene from the wild-type gene present in the community. The forward primer binding site was followed by an 8-bp barcode sequence that was used to mark the origin of the gene (Table 1).

**Table 1:** Different origins for the *sul1* gene.^a^

**Genetic environment**	**In alive host/as extracted plasmid**	**BC-sequence**
pUC19	Alive	AACACATC
pUC19	Extracted	AGGAAACA
pUC19 + Integron	Alive	GGCTTGCA
pUC19 + Integron	Extracted	TGGTTCAA
pUC19 + Transposon	Alive	GGAGTAGA
pUC19 + Transposon	Extracted	CTTCAAAC

^a^The gene was added into the experiment in plain pUC19 or inside pUC19 within an integron cassette or transposon. Each gene in different genetic environments was also added to the experiment either in a live *E. coli* host or as extracted DNA (“dead host”).

Six different barcode sequences were used for six different genetic and physiological origins of the gene. The forward primer binding site and the barcode sequence were added right after the start codon and the reverse primer binding site was added upstream of the stop codon. The gene’s function with primer binding sites and barcodes was tested with sulphamethoxazole MIC test strips (Liofilchem, Italy) in pUC19 in *E. coli*. Each barcode together with the primer binding sites was tested against a strain with a wild-type *sul1* gene and they all conferred resistance similarly to the wild-type gene.

**Figure 2:**
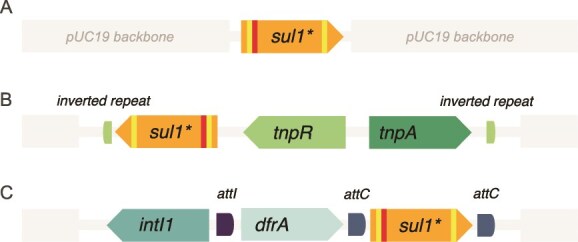
The genetic environments used in the experiment. (A) Gene in non-mobilizable plasmid. The modified *sul1* gene replaced the *lacZ* gene in pUC19 and uses its promoter. (B) Gene inside the non-mobilizable plasmid inside transposon. The transposon included inverted repeats, the modified *sul1* gene replacing the *bla* gene and using its promoter, *tnpR* resolvase, and *tnpA* transposase genes. (C) Gene in the same non-mobilizable plasmid inside an integron. The integron consisted of an integrase *IntI1*-gene, original first cassette (*dfrA*), with the second cassette replaced with our *sul1* gene. The insert ended after the second attC site.

For the first origin a pUC19 plasmid was used (Fig. 2A). The *lacZ* gene was replaced with the modified *sul1* gene from the start codon to the stop codon so that the *lacZ* promoter would work as a promoter for the *sul1* gene. For the other origins, the insert was added into the pUC19 plasmid SmaI restriction site. For the second origin, the *sul1* was added inside a transposon sequence (sequence copied from Tn1 in RP4 plasmid, NCBI: BN000925 [31]) where the *bla* gene inside the transposon was replaced from the start codon to the stop codon with the modified gene (Fig. 2B). For the last origin, part of an *intI1* integron (sequence copied from a pKJK5 plasmid, NCBI: AM261282.1 [32]) was added and the putative spectinomycin resistance gene (second cassette) *aadA11b* was replaced with the *sul1* from the start codon to the stop codon (Fig. 2C). The constructs were introduced into their *E. coli* host with electroporation (0.1-cm cuvette, 2 kV, 200 Ω, 25 μF). Each of the genetic environments had two versions with different barcodes inside the gene. One of them was added to the community inside the living *E. coli* host, and the other one was extracted and the naked DNA was used to represent a dead host and thus to see whether transformation could be detected. The cells were grown similarly before the experiment. The transposon and integron inserts were synthesised and the plasmids were constructed by Genscript (Netherlands). The sizes of the different constructs were 3.3 kbp for pUC19 alone, 5.7 kbp for pUC19 with the integron, and 7.7 kbp for the pUC19 with the transposon.

Because pUC19 is a non-mobilizable plasmid, two conjugative plasmids, pKJK5 and RP4, were also added into the community to enable further spread of the genes. Similarly to the pUC19 constructs, the conjugative plasmids were added inside the living *E. coli* host. This addition allowed the possibility for the genes inside the MGEs to change their genetic environment from their original plasmid to the conjugative plasmids, should they end up in the same cell. All of the plasmids were originally inside their host separately. The pKJK5 plasmid harbours the wild-type *sul1* gene, which is otherwise absent from at least those members of the community that were previously fully sequenced [33].

### Experimental treatments

The experiment included five antibiotic and three temperature treatments, with each combination replicated five times. Two antibiotics, sulphamethazine (Thermo Scientific) and tetracycline (Sigma), were chosen to match the resistance conferred by the modified *sul1* gene and the tetracycline resistance gene (*tetA*), which was present in both of our conjugative plasmids. To determine selective, but below MIC, values for the antibiotics in the experiment a broth microdilution experiment was conducted in R2A broth (Neogen Culture Media, NCM0188A) at 25°C. The growth was measured as absorbance with optical density at 600 nm (OD_600_ [Infinite M200, Tecan]) so that values above 0.06 were considered to indicate growth and we used the first clear drop in otherwise stable absorbance as an estimate for minimal selective concentration (MSC) (results in Table S2). Because the values varied quite a lot, two concentrations of each antibiotic were used (0.02 and 0.2 μg/ml for tetracycline and 2 and 20 μg/ml for sulphamethazine) to keep levels above the MSC but below the MIC for more species.

The experiment was carried out at three different temperatures. The lowest temperature was 15°C, which would still allow some growth, and the highest was 37°C, which is the optimal temperature for many human-associated species. A middle temperature of 25°C was chosen.

### Experiments in microcosms

#### Preparing the community

To test our barcode tracking system, we conducted a 20-day microcosm experiment in which the modified genes were allowed to spread naturally. To begin the experiment, all of the bacteria were grown separately in R2A medium at 25°C for 1–4 days until turbid (depending on the species). The host *E. coli* strains and the receiving community were mixed separately (9 ml of each *E. coli* strain or 2 ml of each receiving community strain) and the mixtures were centrifuged at 15 000 *g*, and 4°C for 1 minute and resuspended to M9 minimal medium salts (MP Biomedicals, LLC, cat. 3037–032) solution to OD_600_ = 0.2. We used 150 μl of both mixtures to start the experiment. The rest was aliquoted and stored at −80°C in glycerol for use as experimental maintenance stock. Aliquots for receiving and donor communities were stored separately.

#### Preparing the microcosm bottles

We used 5.6 ml of R2A broth as the growth medium in 20-ml bottles (scintillation vials, DWK Life Sciences) to allow good aeration. We then added 5 g of washed 0.1–0.2-mm glass beads (SiLibeads® Glass beads Type S, Sigmund Lindner, Germany), which have been shown in previous experiments to increase the probability of HGT in rarer species in the bacterial community [18]. After sterilisation of the microcosm bottles, 40 μl of antibiotic stock (in DMSO) was added to final concentrations of 0.02 and 0.2 μg/ml tetracycline or 2 and 20 μg/ml of sulphamethazine to create the antibiotic treatments. The same volume of DMSO was added to the treatment without antibiotics to control the possible effects of DMSO. The bottles were stored for a maximum of 24 hours at +4°C before using them in the experiment. The 25°C and 37°C treatment bottles were preheated to their target temperature before adding the communities.

#### The experiment

The experiment was conducted at either 15°C, 25°C, or 37°C in 3D rotation with aeration for 20 days. The experiment was maintained by adding 150 μl (2,5%) of communities from old bottles to fresh bottles along with dilutions of donor and recipient community stocks every 96 hours so as not to lose species diversity and the modified genes of interest. The stock dilution was made with 18 ml of ice cold, sterile M9 salts solution and 1 ml of each community stock, and 150 μl of the dilution was added to each bottle. The microcosm bottles were always preheated to the target temperature before adding the communities to keep the temperature as even as possible. After maintaining the experiment, 1-ml samples were taken from old bottles for epicPCR analysis and DNA extraction. EpicPCR samples were stored in 28% glycerol to protect the cells, and the sample for DNA extraction was centrifuged at 16 000 *g* and stored as a pellet. Both samples were stored at −80°C until further analysis.

### Analysis

#### Identifying the carriers of the *sul1* gene with epicPCR

To analyse which species the genes had spread to, the samples stored in glycerol from the final time point were analysed with epicPCR. Primers binding to the primer binding sites were used to target the modified *sul1* gene. To target the 16S rRNA gene, we used 519F (as part of the bridge primer) and 1492R in fusion PCR and 785R in nested PCR (see sequences in Table S3). The epicPCR was carried out as reported in Dekić Rozman *et al*. [23], with the exception that no count chamber was used and cell count was approximated so as not to be too high based on the cell-to-bead ratio. DMSO was added to the fusion PCR to a final concentration of 5%. The amplification results were visualised with gel-electrophoresis (E-Gel EX 2% Agarose, Invitrogen). The samples with amplicon sizes of ~1.2 kbp were considered positive results and sent for sequencing. Because epicPCR is not a quantitative method, those samples for which a band could not be seen on the first run were analysed again with double volume in the first epicPCR step (fusion PCR) to obtain a visible band. For further information about the primers and PCR programs used, see Tables S3 and S4.

#### Community composition and sequencing

For tracking the change of the genetic environment with metagenomes and determining the community composition with 16S rRNA gene amplicon sequencing, the DNA was extracted from frozen cell pellets with the Qiagen PowerLyzer PowerSoil kit. The pellet was resuspended into 250 μl sterile phosphate-buffered saline solution, and half of the volume was added into the kit's PowerBead Tubes. After that the process was continued according to kit instructions from step 2, and the DNA was eluted into 50 μl of the kit elution buffer. For the 16S rRNA PCR primers, 519F and 785R were used (sequences in Table S3, PCR programme in S5). All sequencing was done at the Biotechnical Institute, University of Helsinki, using either the Pacific Biosciences (PacBio) Revio instrument for long reads (epicPCR and metagenome sequencing) or Illumina Miseq for short reads (16S rRNA amplicon sequencing).

### Sequence analysis

#### 16S rRNA amplicons

The 16S rRNA gene sequences were trimmed and processed as described in Dekić Rozman *et al*. [23] using DADA2 v.1.28.0 [34]. Taxonomic assignment was based on the known 16S rRNA gene sequences of the strains used (or species used, if strain information was not available). The 16S rRNA sequence data were normalized by dividing the number of reads for each species by the number of 16S rRNA gene copies of the strains (or species) and the total number of reads per sample to get the relative abundance of each species in each sample. All data analysis was done in R v.4.3.2 [35] with phyloseq v.1.44.0 [36].

#### EpicPCR

The epicPCR amplicon sequencing data from each treatment were further multiplexed based on the barcode sequence using cutadapt v.3.5 [37]. The two parts of the read were split into separate files based on the bridge primer sequence (Table S3) using cutadapt. The validity of each read pair was checked by searching the *sul1* part against the original *sul1* sequence using BLASTN [38]. The 16S rRNA gene part was annotated against the database of 16S rRNA genes of the synthetic community using BLASTN.

### Metagenome analysis

To determine whether the genes had moved from the non-mobilisable pUC19 plasmids to somewhere else in the carrier genomes, we did a PacBio long-read metagenome analysis. The barcode sequences were used to identify and separate reads coming from the different origins with cutadapt v.3.5. Resulting reads were mapped against the different origin environments in pUC19 with minimap2 v.2.24 [39] and further processed with samtools v.1.18 [40]. Mapping results were visualised with IGV v.2.16.2 [41] to find misaligned regions indicative of horizontal movement of the region.

### Statistical analysis

Data were explored for missing values, outliers, collinearity, and proportion of zeros according to Zuur *et al*. [42] To see whether the different treatments had affected the horizontal gene transfer, those results that didn’t imply horizontal gene transfer, i.e., the observations where the gene added into the community inside the living host was observed in *E. coli* were first removed. The remaining observations of gene presence in new hosts was further analysed with PERMANOVA using jaccard distance (vegan v.2.6-4 [43]). The effect of temperature, antibiotic used, their concentration and combined effect of antibiotic and concentration as well as the origin was analysed and the individual bottles were used as strata as the variance differed between them. As the dataset was unbalanced, we calculated the variance from the averages from the levels in each group to define the order of the factors in PERMANOVA. To further test whether different temperatures and origins affected the horizontal gene transfer differently, the Mann–Whitney U test was used (ggpubr v.0.6.0 [44]).

## Results

### Barcode sequences were found and identified

**Figure 3:**
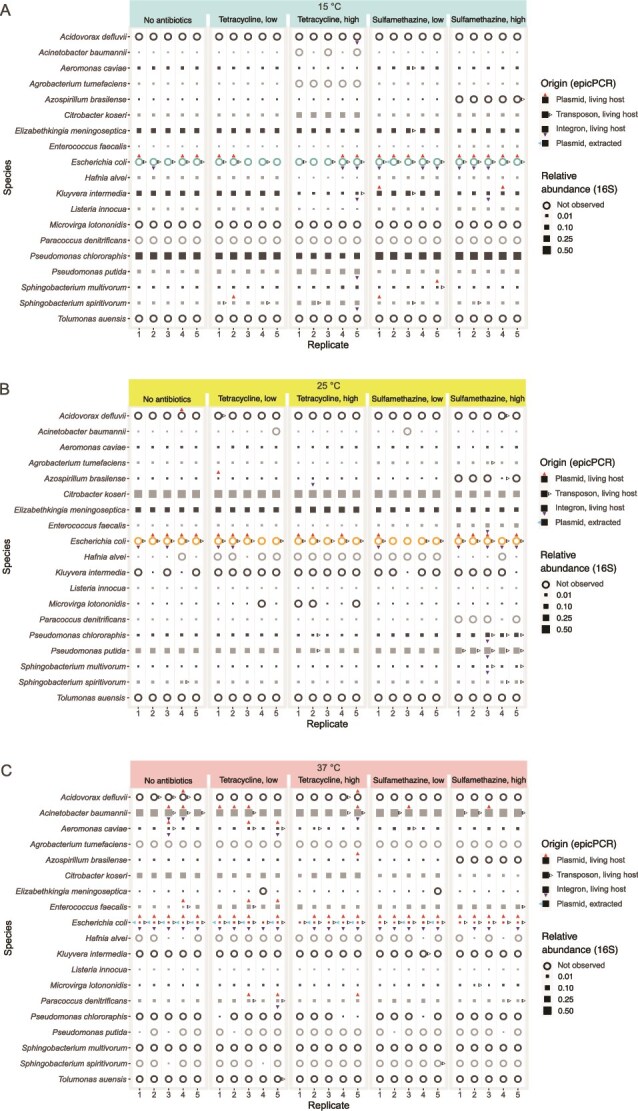
The hosts of the *sul1* genes at the end of the experiment plus the most common species (relative abundance >0.02) in the community. The size of the square shows the abundance of the species in the community while an empty circle indicates that the species was not observed in 16S rRNA amplicon sequencing results. The arrowhead around the abundance indicator shows that the species was carrying the resistance gene in the said treatment–replicate combination. The direction of the arrowhead shows from which origins the gene was detected in the species in presence-absence form. The up-pointing, red arrow stands for the gene in the plasmid originating from a living host; the right-pointing, white arrow for the gene in the transposon originating from the living host; the down-pointing, blue arrow for the gene in the integron originating from the living host; and the left-pointing, light blue arrow for the gene in the plasmid added to the experiment in extracted form (“dead host”). The antibiotic treatments are labelled in the top and replicates in the *x*-axis. *Escherichia coli* Is highlighted with a different colour. Panels show results from (A) 15°C treatments, (B) 25°C treatments, and (C) 37°C treatments.

We were able to distinguish barcodes from our epicPCR and metagenome sequences, and the primer binding sites were detected from the metagenome sequences. Based on the barcodes we could see the origin of the genes and make comparisons between how they had spread and persisted in the experiment communities. The genes originating from transposons, integrons, or plasmids alone, introduced inside a living host, were all detected in the epicPCR sequences under the different experimental conditions. We did not detect the genes originating from the extracted plasmids with transposons and integrons, and the extracted plasmid was found to be the source for the gene in *E. coli* only (Fig. 3). There was considerable variation in the host results between the five replicates, and all five replicates gave identical results only in a few cases.

### The gene had spread to new species

The *sul1* gene had spread in total to 15 new species. Some of them had obtained the gene more often (for example *Acinetobacter baumannii* and *Sphingobacterium spiritivorum*) while others were found to be carrying it only once (for example *Agrobacter tumefaciens* and *Tolumonas auensis*). The most common new host species were *A. baumannii* (21 occurrences), *E. coli* (15 occurrences of extracted plasmid origin), *S. spiritivorum* (12 occurrences), *Aeromonas caviae* (11 occurrences) and *Acidovorax defluvii* (10 occurrences). *A. baumannii* was found to be carrying the gene only at 37°C, and except for one occurrence at 15°C, *E. coli* had taken up the extracted plasmid at the same temperature. The species *S. spiritivorum* was the carrier most often at 15°C, but was found as a host at all different temperatures. The species *A. caviae* and *A. defluvii* had taken up the gene most often at 37°C. Therefore, species seemed to have a preferred temperature (or community composition) for taking up the gene.

Relative community composition was clearly different in different temperatures (Fig. 3, or more clearly in Fig. S6) and small changes were observable with different antibiotic treatments as well. There did not seem to be a connection between the abundance of the species and the species being the host for the resistance gene. The species *A. baumannii* was a common host at 37°C, a temperature where it dominated, but *Citrobacter koseri* was never found as the host, even though it was relatively common at all temperatures. On the other hand, *A. defluvii* never reached the detection threshold for 16S rRNA amplicon analysis, but it was found carrying the gene frequently and at all temperatures. Also, the original carrier, *E. coli*, was not detected at the two lower temperatures with 16S rRNA amplicon analysis but was found in epicPCR results in almost all of the different treatments.

Even though the replicates were similar to each other in terms of relative community composition, the detected spread of the genes was less repeatable. In many cases the gene has spread to a certain new species only once in the five replicates, as in *A. defluvii* at 15°C at higher tetracycline concentrations (Fig. 3A). There are also some treatment combinations for which the gene from a certain origin has spread more frequently, such as in conditions of higher sulphamethazine concentrations at 25°C, for which the gene originating from the transposon had spread to *Pseudomonas putida* in all five replicate communities and to *Pseudomonas chlororaphis* in three communities (Fig. 3B).

Despite our observation of the spread of the gene to new species according to epicPCR results, no change of genetic environment was detected from metagenome sequences. The surroundings of the barcodes always matched those of the experiment in which they were introduced.

### Effect of temperature and origin on the spread

**Figure 4:**
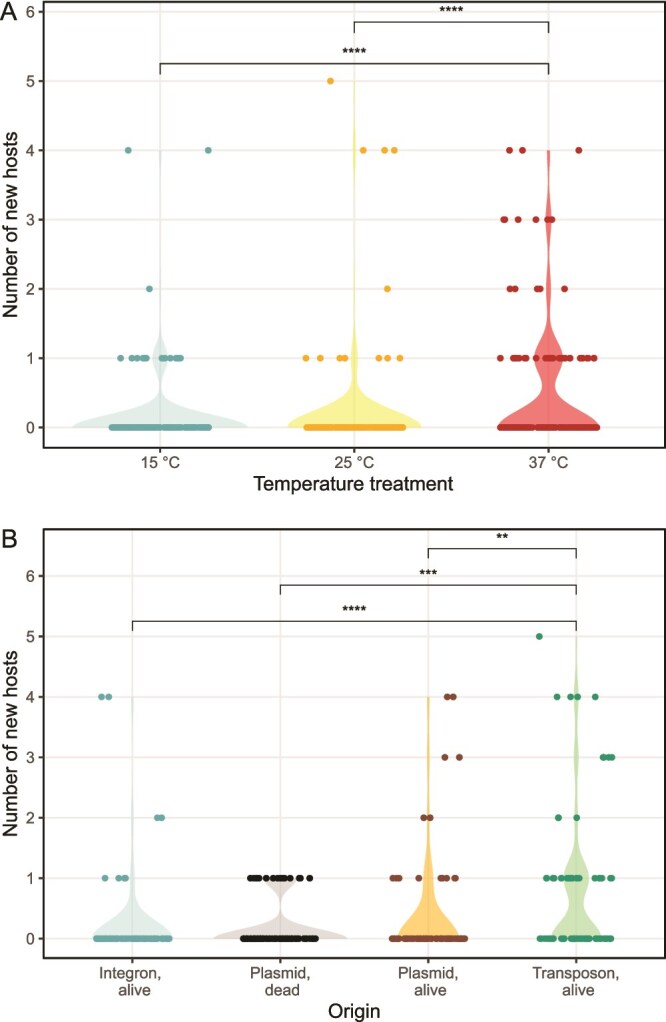
Effects of (A) different temperatures and (B) origins of the horizontal gene transfer of the *sul1* gene. Significance values: ^**^P < .01, ^***^P < .001, ^****^P < .0001.

All the analysed treatments had statistically significant effects on the horizontal gene transfer of the genes (Table 2). However, the effect size was above 0.09 only with temperature and origin (*R*^2^ value, Table 2). When further analysed with the Mann–Whitney U test, only the temperature of 37°C (Fig. 4A) and a genetic environment of transposon originating from the living host (Fig. 4B) differed statistically significantly from the other treatment and origin options.

**Table 2:** PERMANOVA results for the effect of treatments to the horizontal gene transfer.^a^

**Factor**	***P* value**	***F***	***R*** ^**2**^
Antibiotic	.0001	2.3370	0.04314
Concentration	.0001	2.5763	0.02378
Temperature	.0001	10.8203	0.09986
Origin	.0001	9.0053	0.24933
Concentration: antibiotic	.0037	1.2668	0.01169
Residual			0.57220

^a^The *P* values are significant for all treatments, but only temperature and gene origin have higher *R*^2^ values. Altogether the factors explain a bit over 40% of the variation in the data.

## Discussion

In this proof-of-concept study, we have introduced and initially tested a sequence-based barcode system. The idea is to allow the tracking of HGT of antimicrobial resistance of multiple genes or the same gene originating from different genetic environments simultaneously. This tracking together with epicPCR enabled us to see which new species the genes spread to. We were able to use this system to research how different factors affect the movement of the genes, and with the use of barcodes and bioinformatic analysis we were able to see whether their genetic environment changes. In this study we used origin, both genetic and host conditional (alive or dead host) as well as different environmental treatments.

In addition to barcodes, we added artificial primer binding sites (PBSs) inside the genes. This method has several advantages. In the future, analysis of multiple genes may be performed in one run of epicPCR, which minimises the workload. The primer binding sites also help with distinguishing the added genes from the the genes existing in the community naturally and detecting them in the bioinformatics protocol.

We observed that temperature and gene origin could have effects on the horizontal gene transfer of (antimicrobial resistance) genes. We had relatively few observations, which did not allow the use of more detailed statistical analyses such as regression analysis and it also means that some effects, which could eventually play an important part in the whole, could still be hidden. We got some hints that even species rare in the community (such as *A. defluvii* in this case) may play an important part in the movement of genes, because we observed that these species carry the genes rather often despite the species being uncommon or altogether missing from the 16S rRNA amplicon reads. This situation is possible because epicPCR starts from a single cell and provides products only from those species that are carrying the gene of interest, whereas the 16S rRNA amplicon results are compositional [45]. Similar results have been acquired previously with conjugative plasmids, for which HGT does not occur in the most common species [18, 46].

In this study we did not observe any change of genetic environments from our metagenomes, although this would be one of the most interesting occurrences to study with this system. It must be noted that failure to detect movement in the metagenomes does not prove absence of it. The movement might be relatively rare and masked by the high copy number of the pUC19 plasmid used as the original vector. It is also possible that no change of genetic environment happened, but based on our epicPCR results from integron origin, we consider that to be unlikely. We can speculate that, based on how the gene in the integron disappeared in most of the replicates but spread to many new species in some replicates, the gene cassette was transferred to the integron in pKJK5, which allowed it to mobilise more efficiently. But without data to support this possibility, for now, it remains a hypothesis.

Because epicPCR-amplified genes from new hosts barcoded as plasmid only, we believe that the plasmid itself was moving as well. We do not know how, but possible ways are transformation, transduction, and extracellular vesicles. pUC19 has been previously observed to be able to enter extracellular vesicles, potentially enabling the movement between strains of taxa more distant than the other two, which are restricted by species ability to enter natural competence and virus host ranges [47]. The *oriT* area, not the lack of it is sufficient enough to hijack the machinery [48]. Therefore without jumping from pUC19 to pKJK5 or RP4, the gene should not be able to move through conjugation. There are some published data about non-conjugative plasmids moving (for example, between *E. coli* strains [49]) so it does not seem so surprising that the pUC19 would have moved. Conjugation is usually thought to be the dominant mechanism for HGT [50, 51], and much of our understanding of conjugation is derived from studies performed in controlled two-strain mating setups with short times for the transfer to happen. This approach potentially overlooks, for example, possible transformation of bacterial cells with temporary weakened membranes by a plasmid that is released from dying bacterial cells, which could have happened in this study. The 96-hour transfer interval allows more growth stages for HGT to occur, which might make the nonconjugative options more likely.

The most common new host species seemed to have a preferred temperature at which they took up the gene, which may hint that there was something in those conditions that made them biologically more likely to take up the gene. Whether such an outcome is attributable to the temperature, community composition, growth state (as the OD_600_ got lower in 37°C, indicating cell death), or something else in their biology, we do not yet know.

 Because this was, to our knowledge, the first experiment employing a system of this kind, there are additional possibilities for the technical optimisation of the experimental set up. In the future a plasmid with a low copy number should be used, especially if metagenomic analysis is included, so the signal from the new genetic environment is not drowned by the reads from original origin. When researching factors affecting HGT, to get more observations for more robust statistical analysis, conjugative and mobilizable plasmids could be used to ensure higher transfer rates. There is also a challenge in using a sequence barcode: it can mutate, especially in longer experiments. One way around this situation could be to make the barcode sequence longer so that small changes would not make the sequence unrecognisable. In the current study we have already observed some possible point mutations in some of the reads of our 20-day experiment here, although we cannot definitively rule out these observations being sequencing errors.

This system could help bridge the gap between environmental samples and results from the laboratory. Although in our first experiment we used an artificial microbial community and therefore had problems similar to those of other laboratory experiments, these problems may potentially be overcome. The barcode-resistance gene combination allows the researching of many MGEs at the same time in complex communities, and this way the problem of researching the effect of different factors to just one MGE is overcome. With the added sequences in the genes, they can be used in laboratory settings with natural communities that already carry (the same) genes and MGEs of their own. The extra sequences make it possible to separate the added genes from those already in the community. This process can bring us closer to understanding of HGT in the wild, recognizing the factors affecting it and, finally, planning interventions to mitigate the spread of resistance genes and hopefully save lives.

Based on our results, we have created a working barcode system, which together with epicPCR and metagenomics, allows a new way to track horizontal gene transfer of a resistance gene from different genetic origins. This process can be used in complex bacterial communities, which could carry resistance genes of their own. In the future, this method should be tested with multiple resistance genes and more natural communities and MGEs to unlock its full potential.

## Supplementary Material

Supplementary_material_Partanen_et_al_final_ycaf113

Supplementary_figure_S4_ycaf113

## Data Availability

The data for this project are stored in European Nucleotide Archive (ENA) at EMBL-EBI under project number PRJEB85727. Upon article publication the data will be made public and all custom codes used for the analyses will be available from https://github.com/AVKPartanen/Barcodes1.

## References

[1] O’Neill J . Tackling Drug-Resistant Infections Globally: Final Report and Recommendations. Government of the United Kingdom, London, England, 2016.

[2] Murray CJL, Ikuta KS, Sharara F. et al. Global burden of bacterial antimicrobial resistance in 2019: a systematic analysis. *Lancet* 2022;399:629–55. 10.1016/S0140-6736(21)02724-0 35065702 PMC8841637

[3] D’Costa VM, King CE, Kalan L. et al. Antibiotic resistance is ancient. *Nature* 2011;477:457–61. 10.1038/nature10388 21881561

[4] Larsson DGJ, Flach C-F. Antibiotic resistance in the environment. *Nat Rev Microbiol* 2022;20:257–69. 10.1038/s41579-021-00649-x 34737424 PMC8567979

[5] Pang Y, Brown BA, Steingrube VA. et al. Tetracycline resistance determinants in mycobacterium and Streptomyces species. *Antimicrob Agents Chemother* 1994;38:1408–12. 10.1128/aac.38.6.1408 8092846 PMC188220

[6] Séveno NA, Kallifidas D, Smalla K. et al. Occurrence and reservoirs of antibiotic resistance genes in the environment. *Rev Res Med Microbiol* 2002;13:15–27. 10.1097/00013542-200201000-00002

[7] Larsson DGJ, Andremont A, Bengtsson-Palme J. et al. Critical knowledge gaps and research needs related to the environmental dimensions of antibiotic resistance. *Environ Int* 2018;117:132–8. 10.1016/j.envint.2018.04.041 29747082

[8] Smalla K, Cook K, Djordjevic SP. et al. Environmental dimensions of antibiotic resistance: assessment of basic science gaps. *FEMS Microbiol Ecol* 2018;94:fiy195. 10.1093/femsec/fiy195 30277517

[9] Kim D-W, Cha C-J. Antibiotic resistome from the one-health perspective: understanding and controlling antimicrobial resistance transmission. *Exp Mol Med* 2021;53:301–9. 10.1038/s12276-021-00569-z 33642573 PMC8080597

[10] Sandegren L, Linkevicius M, Lytsy B. et al. Transfer of an Escherichia coli ST131 multiresistance cassette has created a Klebsiella pneumoniae-specific plasmid associated with a major nosocomial outbreak. *J Antimicrob Chemother* 2012;67:74–83. 10.1093/jac/dkr405 21990049

[11] Peter S, Bosio M, Gross C. et al. Tracking of antibiotic resistance transfer and rapid plasmid evolution in a hospital setting by Nanopore sequencing. *mSphere* 2020;5:e00525–0. 10.1128/mSphere.00525-20 32817379 PMC7440845

[12] Arredondo-Alonso S, Pöntinen AK, Gama JA. et al. Escherichia coli plasmidome maps the game of clones. *bioRxiv* 2023;2023:2023.10.14.562336.

[13] Fondi M, Karkman A, Tamminen MV. et al. “Every gene is everywhere but the environment selects”: global geolocalization of gene sharing in environmental samples through network analysis. *Genome Biol Evol* 2016;8:1388–400. 10.1093/gbe/evw077 27190206 PMC4898794

[14] Partridge SR, Kwong SM, Firth N. et al. Mobile genetic elements associated with antimicrobial resistance. *Clin Microbiol Rev* 2018;31:10.1128/cmr.00088-17. 10.1128/cmr.00088-17 PMC6148190 30068738

[15] Escudero JA, Loot C, Nivina A. et al. The Integron: adaptation on demand. *Microbiol*. *Spec* 2015;3:10.1128/microbiolspec.mdna3-0019–2014. https://doi.org/10.1128/microbiolspec.mdna3-0019-2014 10.1128/microbiolspec.MDNA3-0019-2014 26104695

[16] Tamminen M, Virta M, Fani R. et al. Large-scale analysis of plasmid relationships through gene-sharing networks. *Mol Biol Evol* 2012;29:1225–40. 10.1093/molbev/msr292 22130968

[17] Klümper U, Riber L, Dechesne A. et al. Broad host range plasmids can invade an unexpectedly diverse fraction of a soil bacterial community. *ISME J* 2015;9:934–45. 10.1038/ismej.2014.191 25333461 PMC4817699

[18] Cairns J, Ruokolainen L, Hultman J. et al. Ecology determines how low antibiotic concentration impacts community composition and horizontal transfer of resistance genes. *Commun Biol* 2018;1:35. 10.1038/s42003-018-0041-7 30271921 PMC6123812

[19] Macedo G, Olesen AK, Maccario L. et al. Horizontal gene transfer of an IncP1 plasmid to soil bacterial community introduced by Escherichia coli through manure amendment in soil microcosms. *Environ Sci Technol* 2022;56:11398–408. 10.1021/acs.est.2c02686 35896060 PMC9387108

[20] Hultman J, Tamminen M, Pärnänen K. et al. Host range of antibiotic resistance genes in wastewater treatment plant influent and effluent. *FEMS Microbiol Ecol* 2018;94:fiy038. 10.1093/femsec/fiy038 PMC5939699 29514229

[21] Daher RK, Filion G, Tan SGE. et al. Alteration of virulence factors and rearrangement of pAsa5 plasmid caused by the growth of Aeromonas salmonicida in stressful conditions. *Vet Microbiol* 2011;152:353–60. 10.1016/j.vetmic.2011.04.034 21621930

[22] Spencer SJ, Tamminen MV, Preheim SP. et al. Massively parallel sequencing of single cells by epicPCR links functional genes with phylogenetic markers. *The ISME Journal* 2016;10:427–36. 10.1038/ismej.2015.124 26394010 PMC4737934

[23] Dekić Rozman S, Puljko A, Karkman A. et al. Bacterial hosts of clinically significant beta-lactamase genes in Croatian wastewaters. *FEMS Microbiol Ecol* 2024;100:fiae081. 10.1093/femsec/fiae081 38796694 PMC11165274

[24] Sakowski EG, Arora-Williams K, Tian F. et al. Interaction dynamics and virus–host range for estuarine actinophages captured by epicPCR. *Nat Microbiol* 2021;6:630–42. 10.1038/s41564-021-00873-4 33633401

[25] Qin H, Wang S, Feng K. et al. Unraveling the diversity of sedimentary sulfate-reducing prokaryotes (SRP) across Tibetan saline lakes using epicPCR. *Microbiome* 2019;7:71. 10.1186/s40168-019-0688-4 31054577 PMC6500586

[26] Pärnänen KMM, Narciso-da-Rocha C, Kneis D. et al. Antibiotic resistance in European wastewater treatment plants mirrors the pattern of clinical antibiotic resistance prevalence. *Sci Adv* 2019;5:eaau9124. 10.1126/sciadv.aau9124 30944853 PMC6436925

[27] MacFadden DR, McGough SF, Fisman D. et al. Antibiotic resistance increases with local temperature. *Nature Clim Change* 2018;8:510–4. 10.1038/s41558-018-0161-6 PMC6201249 30369964

[28] Gullberg E, Cao S, Berg OG. et al. Selection of resistant bacteria at very low antibiotic concentrations. *PLoS Pathog* 2011;7:e1002158. 10.1371/journal.ppat.1002158 21811410 PMC3141051

[29] Jutkina J, Marathe NP, Flach C-F. et al. Antibiotics and common antibacterial biocides stimulate horizontal transfer of resistance at low concentrations. *Sci Total Environ* 2018;616-617:172–8. 10.1016/j.scitotenv.2017.10.312 29112840

[30] Cairns J, Jokela R, Hultman J. et al. Construction and characterization of synthetic bacterial Community for Experimental Ecology and Evolution. *Front Genet* 2018;9:312. 10.3389/fgene.2018.00312 30154827 PMC6102323

[31] Pansegrau W, Lanka E, Barth PT. et al. Complete nucleotide sequence of Birmingham IncPα plasmids: compilation and comparative analysis. *J Mol Biol* 1994;239:623–63. 10.1006/jmbi.1994.1404 8014987

[32] Bahl MI, Hansen LH, Goesmann A. et al. The multiple antibiotic resistance IncP-1 plasmid pKJK5 isolated from a soil environment is phylogenetically divergent from members of the previously established α, β and δ sub-groups. *Plasmid* 2007;58:31–43. 10.1016/j.plasmid.2006.11.007 17306874

[33] Hogle SL, Tamminen M, Hiltunen T. Complete genome sequences of 30 bacterial species from a synthetic community. *Microbiol Resour Announc* 2024;13:e0011124–4. 10.1128/mra.00111-24 38727234 PMC11237487

[34] Callahan BJ, McMurdie PJ, Rosen MJ. et al. DADA2: high-resolution sample inference from Illumina amplicon data. *Nat Methods* 2016;13:581–3. 10.1038/nmeth.3869 27214047 PMC4927377

[35] R Core Team . R: A language and environment for statistical computing. In: R Foundation for Statistical Computing. Vienna: Austria, 2023, 2023.

[36] McMurdie PJ, Holmes S. Phyloseq: an R package for reproducible interactive analysis and graphics of microbiome census data. *PLoS One* 2013;8:e61217. 10.1371/journal.pone.0061217 23630581 PMC3632530

[37] Martin M . Cutadapt removes adapter sequences from high-throughput sequencing reads. *EMBnet J* 2011;17:10–2. 10.14806/ej.17.1.200

[38] Altschul SF, Gish W, Miller W. et al. Basic local alignment search tool. *J Mol Biol* 1990;215:403–10. 10.1016/S0022-2836(05)80360-2 2231712

[39] Li H . Minimap2: pairwise alignment for nucleotide sequences. *Bioinformatics* 2018;34:3094–100. 10.1093/bioinformatics/bty191. 29750242 PMC6137996

[40] Danecek P, Bonfield JK, Liddle J. et al. Twelve years of SAMtools and BCFtools. *GigaScience* 2021;10:giab008. 10.1093/gigascience/giab008 33590861 PMC7931819

[41] Robinson JT, Thorvaldsdóttir H, Winckler W. et al. Integrative genomics viewer. *Nat Biotechnol* 2011;29:24–6. 10.1038/nbt.1754 21221095 PMC3346182

[42] Zuur AF, Ieno EN, Elphick CS. A protocol for data exploration to avoid common statistical problems. *Methods Ecol Evol* 2010;1:3–14. 10.1111/j.2041-210X.2009.00001.x

[43] Oksanen J, Simpson G, Blanchet F. et al. Vegan: Community Ecology Package, Vol. 2022, 2022.

[44] Kassambara A . Ggpubr: ‘ggplot2’ Based Publication Ready Plots, Vol. 2023, 2023.

[45] Gloor GB, Macklaim JM, Pawlowsky-Glahn V. et al. Microbiome datasets are compositional: and this is not optional. *Front Microbiol* 2017;8:2224. 10.3389/fmicb.2017.02224 29187837 PMC5695134

[46] Li L, Dechesne A, He Z. et al. Estimating the transfer range of plasmids encoding antimicrobial resistance in a wastewater treatment plant microbial community. *Environ Sci Technol Let* 2018;5:260–5. 10.1021/acs.estlett.8b00105

[47] Tran F, Boedicker JQ. Genetic cargo and bacterial species set the rate of vesicle-mediated horizontal gene transfer. *Sci Rep* 2017;7:8813. 10.1038/s41598-017-07447-7 28821711 PMC5562762

[48] Ares-Arroyo M, Coluzzi C, Rocha EPC. Origins of transfer establish networks of functional dependencies for plasmid transfer by conjugation. *Nucleic Acids Res* 2023;51:3001–16. 10.1093/nar/gkac1079 36442505 PMC10123127

[49] Maeda K, Nojiri H, Shintani M. et al. Complete nucleotide sequence of Carbazole/dioxin-degrading plasmid pCAR1 in pseudomonas resinovorans strain CA10 indicates its Mosaicity and the presence of large catabolic transposon Tn4676. *J Mol Biol* 2003;326:21–33. 10.1016/S0022-2836(02)01400-6 12547188

[50] Smillie C, Garcillán-Barcia MP, Francia MV. et al. Mobility of plasmids. *Microbiol Mol Biol Rev* 2010;74:434–52. 10.1128/MMBR.00020-10 20805406 PMC2937521

[51] von Wintersdorff CJH, Penders J, van Niekerk JM. et al. Dissemination of antimicrobial resistance in microbial ecosystems through horizontal gene transfer. *Front Microbiol* 2016;7:173. 10.3389/fmicb.2016.00173 26925045 PMC4759269

