# Supplementary material for: Use of sequence barcodes for tracking horizontal gene transfer of antimicrobial resistance genes in a microbial community
Source: ISME Commun. 2025 Jul 10;5(1):ycaf113. doi: 10.1093/ismeco/ycaf113 (PMC12343072; doi:10.1093/ismeco/ycaf113)
Supplement: Supplementary_material_Partanen_et_al_final_ycaf113 [file supplementary_material_partanen_et_al_final_ycaf113.pdf]

# Supplementary material

**Supplementary table S1:** Full list of species in experiment community

| | Strain name | Species name |
|---|---|---|
| 1 | HAMBI 6 | *Pseudomonas putida* |
| 2 | HAMBI 105 | *Agrobacterium tumefaciens* |
| 3 | HAMBI 216 | *Azorhizobium caulinodans* |
| 4 | HAMBI 251 | *Bacillus subtilis* |
| 5 | HAMBI 254 | *Chromobacterium violaceum* |
| 6 | HAMBI 262 | *Brevundimonas bullata* |
| 7 | HAMBI 395 | *Staphylococcus aureus* |
| 8 | HAMBI 403 | *Comamonas testosteroni* |
| 9 | HAMBI 436 | *Enterococcus faecalis* |
| 10 | HAMBI 1279 | *Hafnia alvei* |
| 11 | HAMBI 1287 | *Citrobacter koseri* |
| 12 | HAMBI 1292 | *Morganella morganii* |
| 13 | HAMBI 1299 | *Kluyvera intermedia* |
| 14 | HAMBI 1842 | *Sphingobium yanoikuyae* |
| 15 | HAMBI 1846 | *Acinetobacter baumannii* |
| 16 | HAMBI 1874 | *Sphingobacterium multivorum* |
| 17 | HAMBI 1875 | *Elizabethkingia meningoseptica* |
| 18 | HAMBI 1896 | *Sphingobacterium spiritivorum* |
| 19 | HAMBI 1923 | *Myroides odoratus* |
| 20 | HAMBI 1972 | *Aeromonas caviae* |
| 21 | HAMBI 1977 | *Pseudomonas chlororaphis* |
| 22 | HAMBI 1992 | *Phyllobacterium myrsinacearum* |
| 23 | HAMBI 2159 | *Paraburkholderia caryophylli* |
| 24 | HAMBI 2160 | *Bordetella avium* |

| | | |
|---|---|---|
| 25 | HAMBI 2164 | *Cupriavidus necator* |
| 26 | HAMBI 2316 | *Listeria innocua* |
| 27 | HAMBI 2443 | *Paracoccus denitrificans* |
| 28 | HAMBI 2494 | *Paraburkholderia kururiensis* |
| 29 | HAMBI 2659 | *Stenotrophomonas maltophilia* |
| 30 | HAMBI 2948 | *Psychrobacter proteolyticus* |
| 31 | HAMBI 3172 | *Azospirillum brasilense* |
| 32 | HAMBI 3237 | *Microvirga lotononidis* |
| 33 | DMS12644 | *Acidovorax defluvii* |
| 34 | DMS2094 | *Trichococcus flocculiformis* |
| 35 | DMS9187 | *Tolumonas auensis* |
| 36 | JE2571 | *Escherichia coli* |

**Table S2**: Minimum inhibitory concentration (MIC) and minimal selective concentration (MSC) values for strains in experiment for sulfamethazine and tetracycline.

| **(HAMBI) strain** | **Sulfamethazine MSC** (µg/ml) | **Sulfamethazine MIC** (µg/ml) | **Tetracycline, MSC** (µg/ml) | **Tetracycline, MIC** (µg/ml) |
|---|---|---|---|---|
| **6** | 32.125 | >256 | 0.375 | 4 |
| **105** | 2 | >256 | <0.03125 | 0.046875 |
| **216** | - | - | - | - |
| **251** | 6 | - | 1 | 2 |
| **254** | 1.5 | >256 | 0.140625 | 0.5 |
| **262** | 2 | >256 | <0.03125 | 0.375 |
| **395** | - | - | <0.03125 | 0.03125 |
| **403** | 16.125 | >256 | 0.2625 | 0.5 |
| **436** | 8.5 | >256 | 0.28125 | 2 |
| **1279** | 10 | >256 | 0.0625 | 0.25 |
| **1287** | 36 | >256 | >16 | >16 |
| **1292** | 36 | >256 | 0.09375 | 0.5 |
| **1299** | 33 | >256 | 0.046875 | 1 |
| **1842** | - | >256 | 0.0625 | 0.75 |
| **1846** | 33 | >256 | 0.046875 | 0.25 |
| **1874** | - | >256 | 0.125 | 1.25 |
| **1875** | 20 | >256 | 3 | >16 |
| **1896** | - | >256 | 1 | 8 |
| **1923** | 68 | - | 2.5 | 10 |
| **1972** | - | - | 0.046875 | 0.125 |
| **1977** | 9 | >256 | 0.375 | 8 |
| **1992** | 36 | >256 | 0.75 | 16 |
| **2159** | - | - | - | - |
| **2160** | 4.5 | >256 | - | 2.5 |
| **2164** | 1.5 | >256 | 0.046875 | 0.1875 |
| **2316** | - | - | 0.125 | 0.25 |
| **2443** | 1.125 | >256 | 0.03125 | 0.0625 |

| | | | | |
|---|---|---|---|---|
| **2494** | - | - | - | - |
| **2659** | 12 | - | 0.75 | 1 |
| **2948** | 2.25 | 5 | 0.15625 | 0.1875 |
| **3172** | - | - | 0.75 | 0.75 |
| **3237** | - | - | - | - |
| **DMS12644** | - | - | - | - |
| **DMS2094** | - | 12 | <0.03125 | <0.03125 |
| **DMS9187** | - | - | - | - |
| **JE2571** | - | - | 0.09375 | 0.375 |
| **Average** | 17.64881 | 233.5 | 0.98074597 | 3.16650391 |
| **Median** | 11 | 256 | 0.140625 | 0.75 |

**Supplementary table S3**: primers used in epicPCR.

| Primer | Primer name | Sequence |
|---|---|---|
| Forward, fusion PCR | PBSF1.4* | TGGGTCGTGAGCACCTAG |
| Bridge, fusion PCR | 519F2-PBSR1 | GWATTACCGCGGCKGCTGAGTGTTCTGAGGCTCTGC |
| 16S rRNA gene reverse, fusion PCR | 1492R | GGTTACCTTGTTACGACTT |
| Forward, blocking primer | U519F_block** | TTTTTTTCAGCMGCCGCGGTAATWC |
| Reverse, blocking primer | U519R_Block** | TTTTTTTGWATTACCGCGGCKGCTG |
| Forward, nested PCR | PBSF3.1*** | AGCACCTAGGGTCTCATG |
| Reverse, nested PCR and 16S rRNA amplicon PCR | 785R*** | GACTACHVGGGTATCTAATCC |
| Forward, 16S rRNA amplicon PCR | 519F*** | CAGCMGCCGCGGTAATWC |

*Targets partly start codon
**With C3 spacers in 3’ end
***With Illumina true-seq adapters and C6 aminolinkers at 5’ end (C6 only in epicPCR)

**Supplementary table S4**: PCR programs used in epicPCR. **S4.1** Fusion PCR, **S4.2** blocking PCR and **S4.3** nested PCR. The annealing temperatures were calculated using New England Biolabs tm calculator (fusion PCR) and Thermo Fisher's tm calculator (blocking PCR and nested PCR).

**S4.1** Fusion PCR

| **T** | **t** | **T** | **t** | | **T** | **t** |
|---|---|---|---|---|---|---|
| 80 °C | 10 s | 94 °C | 5 s | | 72 °C | 5 min |
| 94 °C | 30 s | 53 °C | 30 s | x 32 | 4 °C | ∞ |
| | | 72 °C | 1 min | | | |

**S4.2** Blocking PCR

| **T** | **t** | **T** | **t** | | **T** | **t** |
|---|---|---|---|---|---|---|
| 98 °C | 30 s | 98 °C | 10 s | | 72 °C | 5 min |
| | | 66.5 °C | 30 s | x 30 | 4 °C | ∞ |
| | | 72 °C | 30 s | | | |

**S4.3** Nested PCR

| **T** | **t** | **T** | **t** | | **T** | **t** |
|---|---|---|---|---|---|---|
| 98 °C | 30 s | 98 °C | 10 s | | 72 °C | 5 min |
| | | 57 °C | 30 s | x 39 | 4 °C | ∞ |
| | | 72 °C | 1 min | | | |

**Supplementary table S5**: 16S rRNA amplicon PCR program

| **T** | **t** | **T** | **t** | | **T** | **t** |
|---|---|---|---|---|---|---|
| | | 98 °C | 10 s | | 72 °C | 5 min |
| 98 °C | 30 s | 56.5 °C | 30 s | x 15 | 4 °C | ∞ |
| | | 72 °C | 10 s | | | |

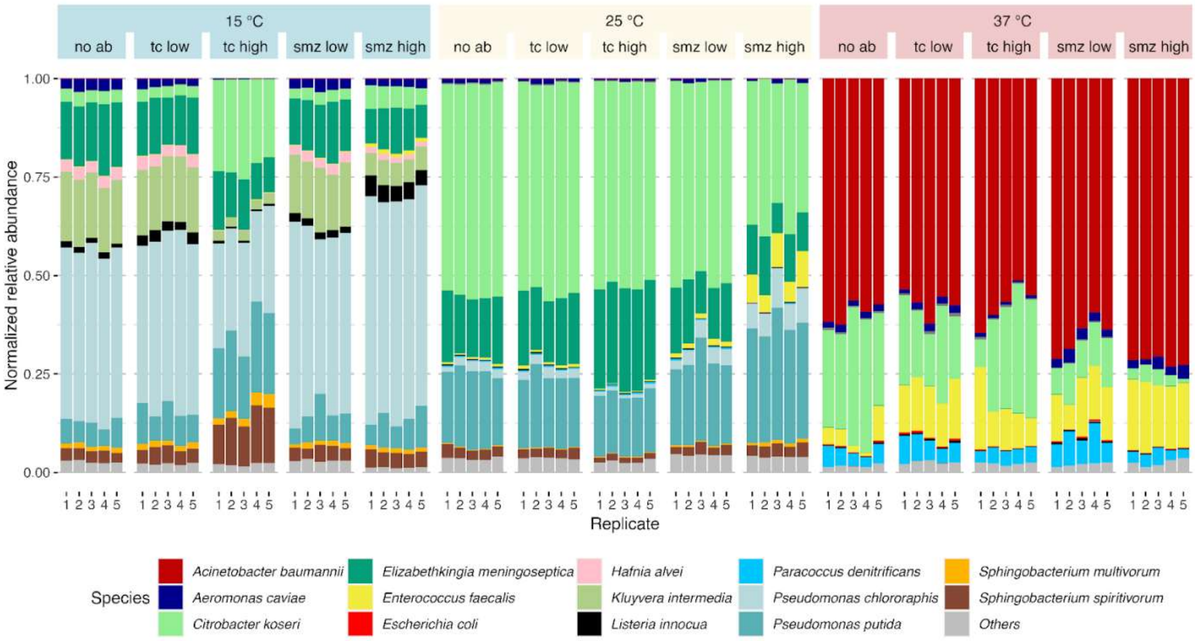


**Supplementary figure S6:** Species abundance based on 16S rRNA amplicon sequencing. Species with always low abundance (relative abundance <0.02) not shown individually (grouped together to “others”) except for the original gene host *E. coli* (only found in very low abundance in 37 degrees). Results normalised to the amount of 16S rRNA gene copies each strain (or species if no information was found on strain level) has and to total number of reads to get relative abundance.

**Table S7:** Number of species for who the experiment treatments were or weren’t between their MSC and MIC. *For tetracycline, we didn’t do measurements as low as 0.02 µg/ml.

| Antibiotic | Concentration (µg/ml) | Species with experiment concentration between MSC and MIC | Species with experiment concentration not between MSC and MIC | MSC and/or MIC not determined or not enough information* |
|---|---|---|---|---|
| Sulfamethazine | 2 | 5 | 8 | 17 |
| | 20 | 11 | | |
| Tetracycline | 0.02 | 0* | 15 | 10 |
| | 0.2 | 11 | | |
