# Supplementary figures and images for: Use of sequence barcodes for tracking horizontal gene transfer of antimicrobial resistance genes in a microbial community
Source: ISME Commun. 2025 Jul 10;5(1):ycaf113. doi: 10.1093/ismeco/ycaf113 (PMC12343072; doi:10.1093/ismeco/ycaf113)

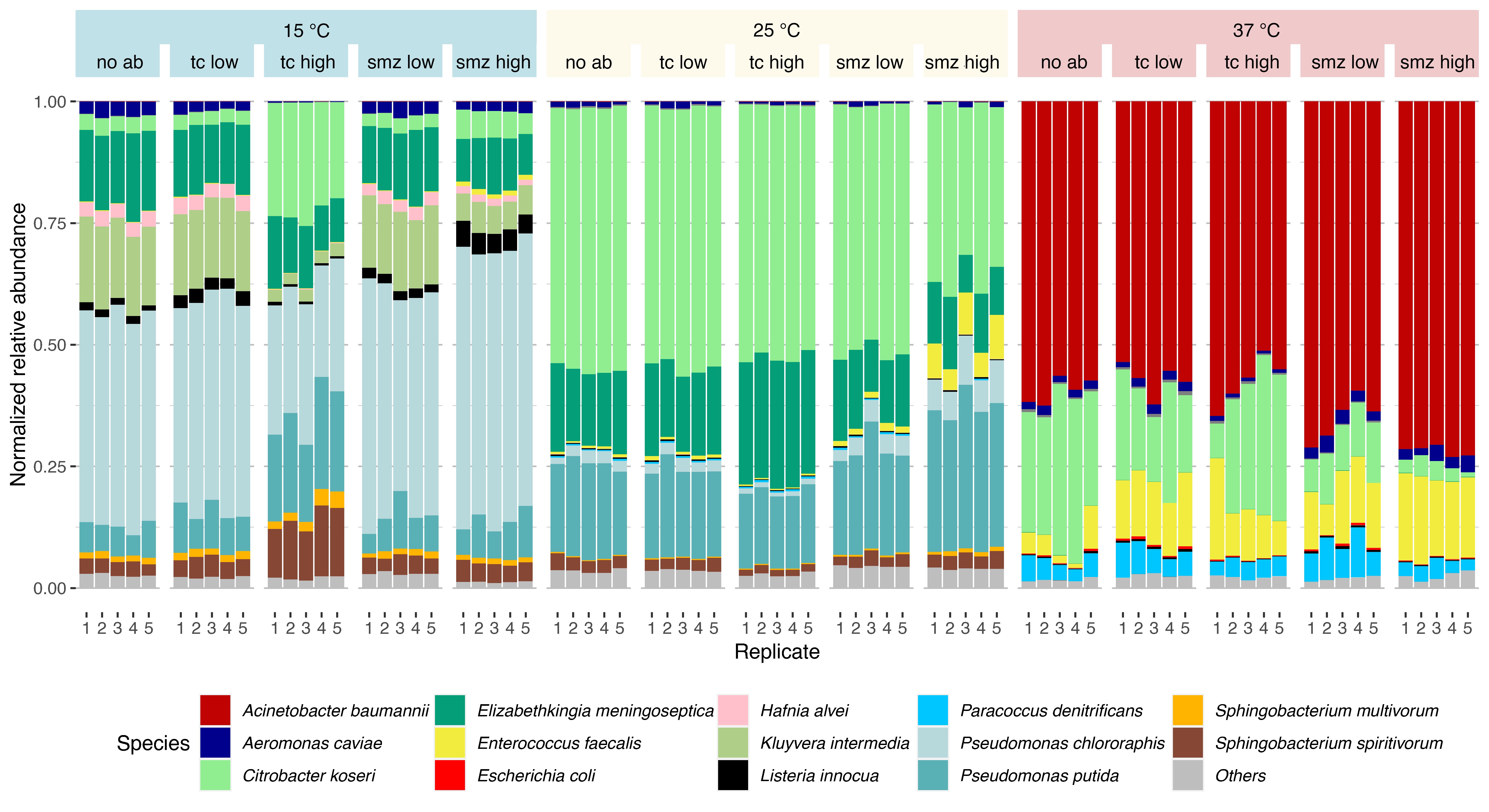

Supplement: Supplementary_figure_S4_ycaf113 [file supplementary_figure_s4_ycaf113.jpeg]
